# Descriptive Cost-Effectiveness Analysis of a Counseling and Coordination Model in Psychosocial Care. Integration of Health Care and Social Rehabilitation

**DOI:** 10.3389/fpsyt.2019.01008

**Published:** 2020-02-12

**Authors:** Anne Berghöfer, Sabrina Hense, Thomas Birker, Torsten Hejnal, Frank Röwenstrunk, Marion Albrecht, Daniela Erdmann, Thomas Reinhold, Barbara Stöckigt

**Affiliations:** ^1^ Institute for Social Medicine, Epidemiology and Health Economics, Charité—Universitätsmedizin Berlin, Berlin, Germany; ^2^ Clinic for Psychiatry, Psychotherapy, and Psychosomatics, Westküstenkliniken Brunsbüttel und Heide gGmbH, Heide, Germany; ^3^ Pflegezentrum Domicil, Heide, Germany; ^4^ Outreach Clinic Heide, Heide, Germany; ^5^ Koordinierungsstelle soziale Hilfen der schleswig-holsteinischen Kreise, Kiel, Germany

**Keywords:** psychosocial care, outreach clinic, cost-effectiveness, service utilization, quality of life, prospective observational study

## Abstract

**Introduction:**

A psychosocial outreach clinic was established to offer counseling and coordination of healthcare and complementary services for persons with psychosocial and mental problems. The cost-effectiveness of these services was measured based on a pre-post comparison.

**Methods:**

A prospective observational study was conducted with clients of the outreach clinic. Data on resource consumption and quality of life were collected at baseline and follow-up after 3, 6, and 12 months using the Client Sociodemographic and Service Receipt Inventory to assess service utilization, and the 12-Item Short Form Health Survey to assess quality of life. The objective of the present analysis was to estimate the relation between monetary expenditure and QALYs (quality-adjusted life-years), before and after the outreach clinic was established, descriptively. The analysis was constructed from payer’s perspective and was supplemented by a sensitivity analysis.

**Results:**

A total of 85 participants were included. Total annual expenditures before the intervention were 5,832 € per client for all service segments. During the 12-months study duration expenditures decreased to 4,350 € including the costs associated with outreach clinic services. QALYs for the 12-month study period were 0.6618 and increased about 0.0568 compared to the period before.

**Discussion:**

Despite methodological limitations due to small sample size, a pre-post comparison and the retrospective cost data collection, this study suggests acceptability of the outreach clinic as cost-effective.

**Conclusion:**

The activities of the outreach clinic as an integrated care model seem to be cost-effective regarding the relation between monetary expenditures and clients’ quality of life.

## Introduction

The German health care system is characterized by a separation into various sectors of outpatient and inpatient acute care, rehabilitation, integration assistance, and social support (1, 2). Psychosocial care is based on several Social Insurance Codes and different payers within these sectors (3). This complex structure makes it difficult for persons seeking psychosocial care to identify services that might be relevant for their individual situation and benefits they are entitled for. As a consequence, a considerable amount of clients in need of psychosocial support receives psychiatric care (with long waiting times for ambulatory treatment or acute inpatient care), leading to unnecessary institutionalization and stigmatization (4, 5).

Structures of case management or outreach clinics that help navigating through the psychosocial and health care system are only available in limited schemes of care in Germany and do not cover the whole population (6–8). Apart from this legal restriction, such services are extremely rare in the German health care system.

In a rural administrative district in the German Federal State of Schleswig-Holstein a psychosocial outreach clinic was established by the management of the administrative district in cooperation with the major provider of inpatient mental health care, a clinic run by the district’s local authority (9). The aim was to offer individual counseling and coordination of healthcare and complementary services for persons with mental and psychosocial problems in the region in order to avoid inappropriate institutionalization in psychiatric inpatient care or supported housing. According to the proposal by Steinhart and Wienberg (10, 11) the model allows for a cooperation of medical and psychosocial care and overcomes the separation by the two Social Insurance Code Books V and XII. While services covered by Code Book V include all forms of acute and long-term medical care Code Book XII comprises assistance for living in institutions, sheltered housing, assistance for employment and day structuring, and measures to participate in the community. The model is based on several years of close cooperation between the hospital and the integration assistance services of the district (12).

Mental health care in the region is financed using a fixed lump sum budget since 2008 (Regional Psychiatry Budget, RPB), which covers various services within a network of providers of psychosocial care (13). Provision of care is committed to a social psychiatric treatment concept, offering day care facilities and walk-in clinics to avoid unnecessary inpatient stays.

The providers of integration assistance and supported housing are reimbursed by the administrative district based on a fee-for-service principle. Since the supply of these services is not budgeted and the market is not regulated, private providers of different services have successfully widened their share of the market over the last years and secured profitable funding for an increased supply of institutionalized care, which is not always in line with the actual supply requirements (4, 14). Therefore, a second aim of the psychosocial outreach clinic was to contain costs of the public authorities that result from an oversupply in the integration assistance sector.

The aim of this study was to estimate the cost-effectiveness of the counseling and coordinative activities of this outreach clinic. Primary endpoint was the ratio between the pre-post change in clients’ quality of life and costs of service utilization in all relevant Social Insurance Code Books.

## Materials and Methods

### Study Design

The study was performed within a multi-part project using a mixed-method approach. The overall project consisted of a) a study with secondary data analyses of routine administrative data (15), b) the prospective observational cost-effectiveness analysis, on which this paper focuses, and c) a qualitative study on consumer and provider satisfaction (9, 12). In this paper only data of the prospective observational cost-effectiveness analysis will be presented. Ethical approval for all study parts was obtained from the responsible ethics committee [Ref. no.131/14 (I)].

In a prospective observational study the utilization of services and quality of life of clients whose treatment and counseling needs were coordinated by the outreach clinic were assessed at the date of first contact with the center (baseline) and followed up after 3, 6, and 12 months. Baseline data were assessed between January 2015 and May 2015.

### Study Participants

The study included all persons (hereinafter referred to as clients, since a psychiatric diagnosis was possible but not necessary for inclusion) aged 18 years and older, who were mentally and linguistically capable to comply with the study requirements, provided written consent (if individual care support was needed, consent of the caregiver was required as well), were resident in the administrative district, and who presently had psychosocial problems and/or a diagnosis of a mental disorder. Persons with severe general illnesses, such as currently treated cancers or progressive neurological diseases were excluded from the study.

Participants could withdraw from the study at any time either retrospectively (this implied the deletion of all previously collected data) or prospectively (no further data were collected).

### Data Collection and Instruments

The clients’ resource utilization of health and social services was assessed with the German adaption of the Client Sociodemographic and Service Receipt Inventory (CSSRI-D) (16, 17). This questionnaire has been designed for people with mental disorders and comprises six categories of assessment: sociodemographic data (age, sex, marital status, ethnic origin, native language, level of education), usual living situation, accommodation details, employment and income, and use of services referring to different Social Insurance Code Books. The instrument was adapted according to the range of services in the administrative district of the study region. The utilization of health and social care services was surveyed retrospectively at each examination date. For baseline, the past 6 months were surveyed, for the further follow-up appointments the utilization between the current and the last follow-up appointment was surveyed, in order to achieve a complete recording of the utilization over the 6 months before the start of the study and the 12 months under study conditions. Clients’ quality of life was assessed with the 12-Item Short Form Health Survey (SF-12) (18). This non-disease-specific questionnaire results in two summary scores: the physical (including subdomains on general health, physical functioning, physical role functioning, bodily pain; PCS) and the mental (including subdomains on emotional role functioning, vitality, mental health, social functioning; MCS) component summary score. The SF-12 allows and is commonly used for index utility calculations as a basis for further analyses on the cost-effectiveness of interventions (19).

Both questionnaires were implemented as face-to-face interviews conducted by trained interviewers.

All data were collected in the outreach clinic where resources to inform the clients about the study, obtain consent and conduct the interviews were provided.

Data collection was performed according to standard operating procedures and adherence to the protocol was assured by quality control site visits conducted by the project lead.

### Data Analysis

The primary outcome of the present analysis was the incremental relation between monetary expenditure from a payer’s perspective and QALYs (quality-adjusted life-years) before and after the outreach clinic services were established.

Therefore a stepwise expenditure calculation was conducted based on information from the CSSRI-D questionnaire. In a first step, the amount of services and resources used in the different service areas as reported by the client was analyzed. Secondly, the expenditure for each service used was then determined based on remuneration and pricing indexes applicable for the respective area of service (20–25). The underlying unit cost is reported in **Table 1**. The reported amount of services used was multiplied with these unit cost assumptions in order to calculate the total expenditure per service area and per client before implementation of the outreach clinics as well as over the study period and for each of the follow-up intervals.

**Table 1:** Major service utilization items in health and social care and allocated cost units in EURO.

Item	Cost (EURO)	Source
Psychiatric inpatient treatment (cost per day)	386	Local clinic
Somatic inpatient treatment (cost per day)	576	[20]
Day-care treatment (cost per day)	168	Local clinic
Outpatient treatment by psychiatrist (cost per visit)	45	[20]
Outpatient treatment by primary care physician (cost per visit)	20	[20]
Sheltered workplace (cost per day)	48	Administrative district
Occupational therapy (cost per unit)	38	[20]
Physical therapy (cost per unit)	16	[20]
Day-structuring measure (cost per unit)	33	
Contact and counseling center (cost per visit)	29	Local service provider
Home nursing (cost per visit)	22	Cost agreement for home nursing between providers and insurer
Police contact	62	State ordinance on administrative fees

**Figure 1:**
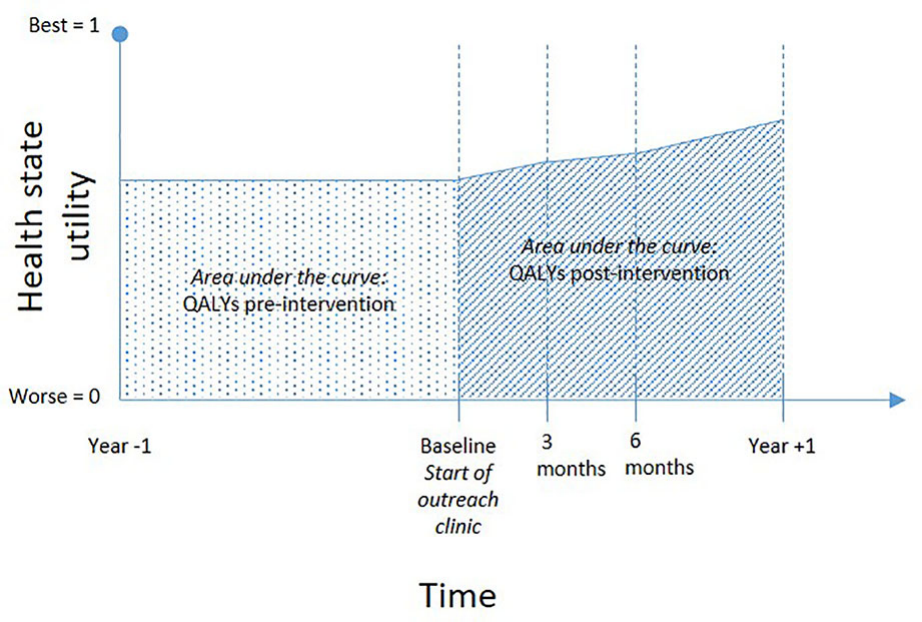
Explanation of the area-under-the-curve method to show changes in quality adjusted life years in the sample under observation.

QALYs were used to get information on effectiveness changes. QALYs relate the quality to the quantity of life lived and are a commonly used measure in health economic studies to assess the non-monetary benefit of interventions. Health state utilities were used as the basis for the estimation of QALYs. The higher the utility value (between 0 and 1), the lower the subjectively perceived health impairment. Health state utilities were calculated for each date of survey (baseline and three follow-ups) by converting the SF-12 quality of life (QOL) data into SF-6D using an algorithm published by the University of Sheffield (26). For the SF-6D, preference weights are available, on which the derivation of health state utilities in our analysis was based on. These utilities were included in the estimation of QALYs by calculating the area under the curve, assuming a linear change between the survey dates. QALYs calculated for the year before the intervention starts are based on the health state utility measured at baseline, which was assumed to be stable during the year before (**Figure 1**).

For measuring the cost-effectiveness, for both values (individual costs, individual QALYs) a pre-post difference was calculated according to the following formulas:

$$\text{Effect differences}:\Delta \text{QALYs }=\text{ QALY}{\text{s}}_{\text{post intervention}}–\text{ QALY}{\text{s}}_{\text{pre intervention}}$$

$$\text{Cost differences}:\Delta \text{Total costs }=\text{ Total cost}{\text{s}}_{\text{post intervention}}–\text{ Total cost}{\text{s}}_{\text{pre intervention}}$$

Both differences were plotted into a cost-effectiveness diagram.

The calculation of SF-12 values for the physical and mental component summary scores followed the analysis algorithm also used by Bullinger and Kirchberger (27).

Missing values in the cost assessment during the follow-up period were imputed by carrying the cost of the last complete follow-up period of the individual patient forward. Missing values in the quality of life assessment were imputed by carrying the last observation of quality of life forward until follow-up after 12 months.

An additional bootstrap analysis with random 1,000-fold resampling of the original population was performed to determine to what extent the results may vary due to many replications of the study. This analysis accounted for the heterogeneity around all health care resource consumption observed in the study. These bootstrapped cost-effectiveness results were additionally used to generate a cost-effectiveness acceptability curve, which show the probability of cost-effectiveness for different threshold values with regard to willingness to pay for one extra QALY (28).

## Results

Overall, 85 clients received counseling and were included in the analysis. Early dropout was low and 73 (85.9%) clients completed the 12-month follow-up. Mean age at baseline was 45.7 (SD 17.0) years, ranging from 18.4 to 77.8 years. About three quarter of the study population was female (**Table 2**). The proportion of clients with an education of less than 10 years was 55%, and 60% were either pensioned or unemployed. More than 50% lived alone and received either social security benefits or were financially supported by their family.

**Table 2:** Characteristics of the study population at baseline.

	N = 85	%
Age (Mean years ± SD; range)	45.7 ± 17.0; 18.4-77.8	
Female sex Male sex	63 22	74.1 25.9
Highest education		
Less than 10th grade	47	55.3
10th grade or higher	38	44.7
Usual living situation		
Living alone (with or without children)	44	51.8
Living with spouse or partner (with or without children)	27	31.8
Living with parents, relatives or other persons	14	16.5
Employment		
Self-employed	16	18.8
Pensioned	16	18.8
Unemployed, unable to work	35	41.2
Other (vocational training, household, etc.)	18	21.2
Monthly net income (Mean Euro ± SD; Range)	669 ± 661; 0-2,700	
Main source of income		
Salary/Pension	39	45.9
Social security benefit, family support	46	54.1

Every individual study participant had used any form of psychosocial service in the 6 months before the first contact with the outreach clinic. However, only 26% of the clients had previously received inpatient psychiatric care.

Health related and complementary expenses (including inpatient, day-care, outpatient, and complementary social care) in the 6 months before first contact with the outreach clinic amounted to an expenditure of 247,842 € in total and 2,916 € per client. Extrapolated to a 12-month period prior to baseline, this resulted in a total estimate of 495,683 € and mean costs of 5,832 €, respectively (**Table 3**). Before baseline, inpatient care accounted for 43%, day-care for 11%, outpatient care for 10%, medication for 12%, and complementary services (including e.g. sheltered workshops, outpatient ergo-therapy, contact or counseling services, support groups) for 19% of the costs. In the 12 months after baseline the total expenditure decreased to 369,780 € over all service areas, with mean costs of 4,350 € per client (**Table 3**). The distribution of expenses over the service areas changed considerably: the proportion of costs for day-care was reduced to nearly 0%, inpatient care cost decreased to 38%, while costs for outpatient care increased about 8 percentage points up to 18%. Complementary care proportion remained rather stable. Overall, the total annual expenditure for clients of the outreach clinic was markedly lower (about one third) after counseling by the outreach clinic than before. In particular, a reduction in inpatient care and complementary costs as well as a complete decline of day care costs was observed.

**Table 3:** Mean costs and 95% confidence intervals (CI) in EURO per user and per client, and total costs before and after counseling and coordination services in outreach clinic.

Observation period	12 months before counseling mean, EURO (CI)				12 months after counseling mean, EURO (CI)				Difference (after intervention minus before intervention)
Cost item	n (user)	Costs per user	Costs per client (n = 85)	Total costs	n (user)	Costs per user	Costs per client (n = 85)	Total costs	Total costs
Inpatient care	22	9,717 (3,208-16,226)	2,515 (682-4,348)	213,774	20	7,043 (2,005-12,080)	1,657 (377-2,938)	140,853	- 72,921
Psychiatric day care	4	13,530 (5,031-22,029)	637 (-22-1,295)	54,121	1	434 (-)	5 (-5-15)	434	- 53,687
Outpatient care	70	713 (533-893)	587 (428-746)	49,914	74	922 (664-1,180)	802 (569-1,036)	68,204	18,290
Medication	53	1,167 (753-1,581)	728 (445-1,011)	61,866	67	928 (627-1,229)	731 (482-981)	62,176	310
Complementary care	75	1,230 (795-1,666)	1,086 (693-1,478)	92,276	73	968 (667-1,270)	832 (563-1,100)	70,696	- 21,580
Other contacts	31	766 (302-1,229)	279 (98-461)	23,733	44	430 (180-679)	223 (88-358)	18,917	- 4,816
Counseling at the outreach clinic	–	–	–	–	85	100	100	8,500	8,500
Overall costs	85	5,832 (3,710-7,953)	5,832 (3,710-7,953)	495,683	85	4,350 (3,000-5,701)	4,350 (3,000-5,701)	369,780	- 125,904

Inpatient care includes hospital treatment for all reasons incl. psychiatric; outpatient care includes visits at primary care, medical specialists, psychotherapy, physician assistants; complementary care includes social assistance, services for rehabilitation and participation in working life and in the community, regular counseling at integration service provider; other contacts include caregivers’ work, household care, legal assistance.

Quality of life was assessed at baseline and at each of the three follow-up points. Summary scores and utilities over all time points are given in **Table 4**. The mental component summary score increased considerably and clinically relevant during the study period as well as the health state utilities. Albeit less pronounced, a rise was also seen for the physical component summary score. QALYs based on utilities referring to the SF-12 results were 0.6618 for the entire 12 months study period compared to only 0.6050 for the period before. Accordingly, the QALYs gained over 12 months due to counseling by the outreach clinic were 0.0568.

**Table 4:** Physical and mental component summary scores and health state utilities for all clients at baseline and each of the follow-ups [(mean and 95% confidence intervals (CI)], missing values imputed by last observation carried forward.

	Baseline	Follow-up 1	Follow-up 2	Follow-up 3
SF-12 physical component summary score	42.9 (40.3-45.5)	43.7 (41.2-46.3)	44.2 (41.6-46.8)	44.6 (42.1-47.1)
SF-12 mental component summary score	35.4 (32.8-38.0)	40.3 (37.7-42.9)	41.2 (38.3-44.1)	44.2 (41.7-46.7)
Utilities	0.6051 (0.5789-0.6314)	0.6535 (0.6263-0.6807)	0.6602 (0.6290-0.6915)	0.6927 (0.6621-7233)

**Figure 2:**
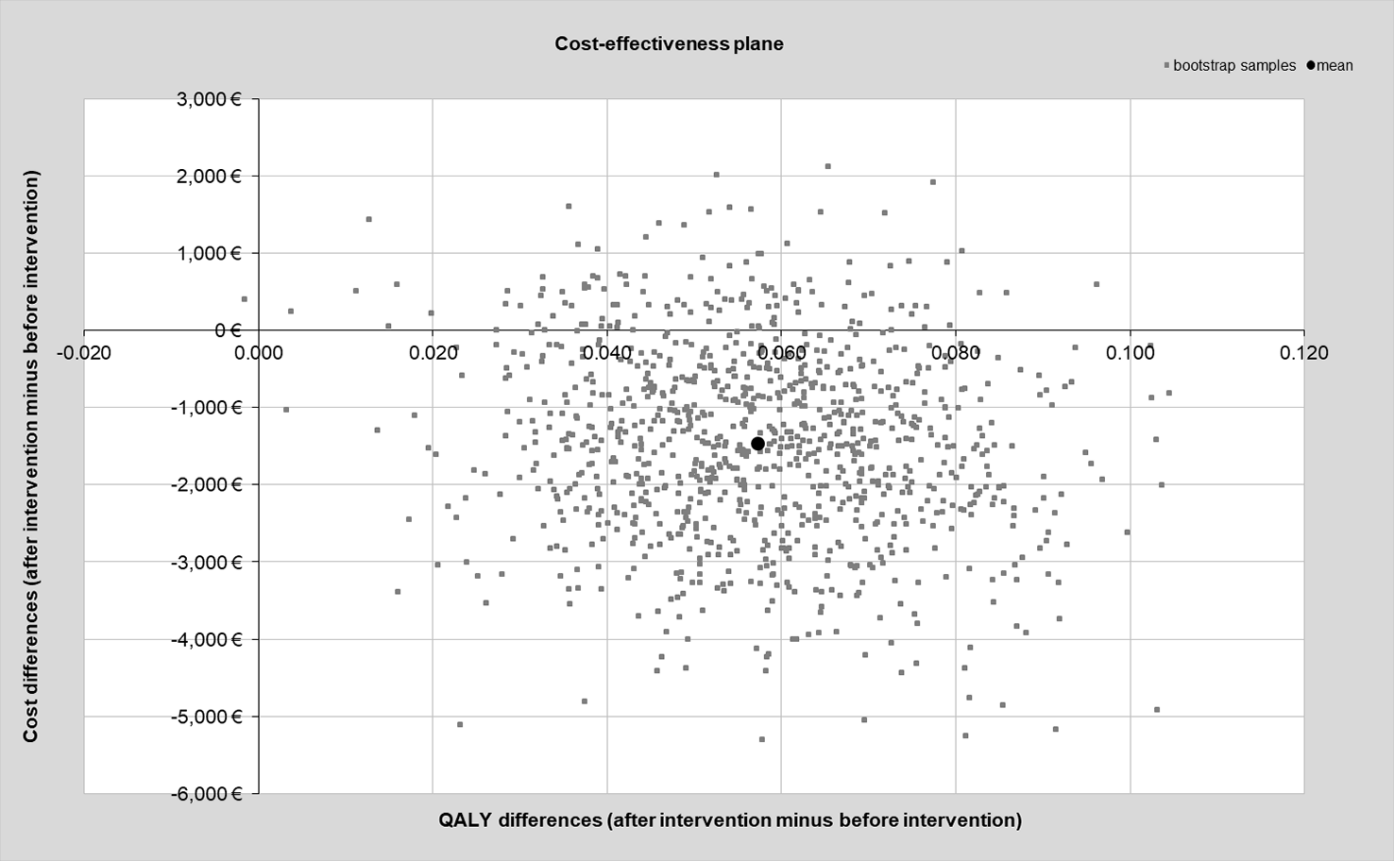
Cost-effectiveness plane of bootstrapped study samples.

After combining the pre-post differences for costs and QALYs, the services of the outreach clinic seem to be cost-effective, since a relevant cost reduction and a gain in QALYs was observable after counseling. This principal result was also proved in the sensitivity analyses, where most of the replicated bootstrap samples are located in the lower right quadrant of the cost-effectiveness plane (**Figure 2**).

**Figure 3:**
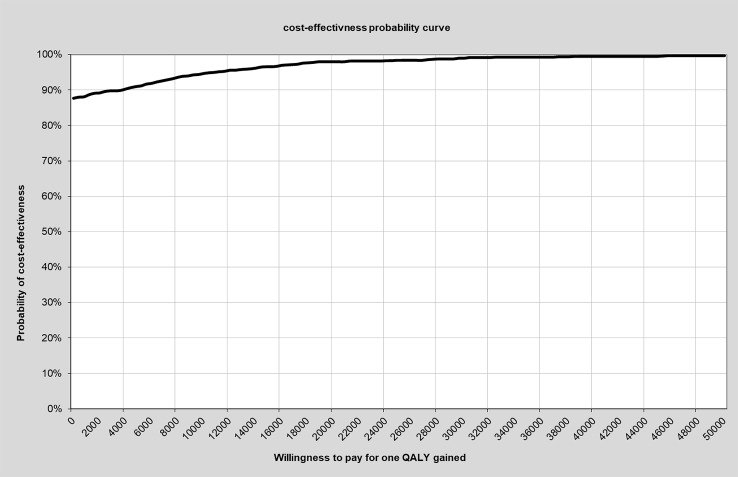
Cost-effectiveness probability curve in relation to willingness to pay for one additional QALY. QALYs, quality-adjusted life-years.

The probability of cost-effectiveness was comparable high, reaching a probability of 99.7% for a willingness to pay for one QALY gained of 50,000 € (**Figure 3**).

## Discussion

Overall, the concept of the outreach clinic appeared to be cost-effective as the clients’ quality of life increased over the study period with a concomitant decrease in costs of medical and psychosocial service utilization over all Social Insurance Code Books.

The non-randomized selection of the study population as well as the lack of a control group clearly lowered the evidence level of the study. The participants were no representative sample of inhabitants of the region in need of care but a cohort of clients that presented themselves at the outreach clinic during the period of recruitment. However, the socio-economic pattern of the study population reflected the general socio-economic structure in the administrative district: the district has been reported to have the lowest population in terms of numbers and density as well as the by far highest proportion of social benefits recipients and unemployment rate in the Schleswig-Holstein region (29). Although the lack of representativeness limits a transfer of the results to the entire region, the analyses allow for first indicative observations on the effect of the outreach clinic’s activities. Albeit a control group of comparable persons that did not receive but would have been eligible for counseling by the outreach clinic would have been desirable, this was not possible due to strategic and logistical reasons in this area of routine care.

Furthermore, the retrospective assessment of data on the utilization of services might have led to an inaccurate recall of information by the clients. To conduct a prospective study, however, would have been possible only with enormous efforts and/or by linking routine data from different sources—which is legally and logistically difficult to realize. As the recall period of 6 months is surveyable, we estimate a possible recall bias as low.

Another limitation is a certain impreciseness and generalization in the allocation of costs. In fact, the assignment of pay rates to the respective services used was based on pertinent remuneration indexes or—if not available—on approximations. This approach is commonly used in healthcare economics and breaks down personnel and operating costs of an institution to the amount of services provided or to an hourly rate, which is then used as an approximation (17, 30). Also, the clients’ reported use of services does not necessarily have to be identical with the expenditures reported by the services to the payer. The actual remuneration might be higher, as not all approved services might ultimately have been made use of by the clients. This discrepancy arising from different perspectives of health economic analyses is though inherent to all health economic studies and needs to be considered when interpreting respective results. Since, however, the cost-effectiveness in the present study could clearly be allocated to the dominant quadrant in the cost-effectiveness plane and a potential under-estimation would apply to the time before as well as after baseline, this was not expected to fundamentally impact on the study results.

People coming to the outreach clinic can be expected to suffer from a high degree of psychological strain, which usually improves after counseling or any other form of intervention. Therefore, a potential regression-to-the-mean effect should be considered regarding the observation of lower costs after counseling compared to the expenditure before counseling—although this comparison was not in the focus of the study. The same applies to the quality of life, which might have been low during the whole year prior to counseling and might increase in the sense of a regression-to-the-mean effect.

The cross-sectoral service of the psychosocial outreach clinic achieves its benefit against the background of the complex structure of the German health system. Therefore, the results cannot be transferred without restriction to other health care systems in which a stronger integration of medical and social work may already be possible.

Finally, it may have come to changes in the surrounding conditions during the period covered by the collected data. These changes may include the provision of health services (e.g. number of physicians in private practice) or in the social environment (e.g. level of unemployment in the region).

Because of this fundamental limitation of a pre-post comparison, the results can only be preliminary and should be confirmed by other study designs. In health care research, however, gold standard methods often cannot be applied because control group and randomization cannot be implemented in everyday health care and because blinding of complex health care models is not possible (31).

A notable strength of the present study was the collection of data with a questionnaire that was specifically adapted to the region and its services. This instrument allowed for a detailed assessment of all types of expenditure related to the clients’ psychosocial conditions. This distinguishes the present study from many other health care economic evaluations. Usually, expenditures are considered exclusively for the sector of statutory health insurances and rehabilitation providers. Social welfare sector expenses are often lacking. This expenditure area though represents a significant cost component with regard to psychosocial and especially psychiatric care. Our analysis therefore creates a more complete picture of respective and relevant expenses.

Although the quality of life increased considerably and clinically relevant during the study period especially in the mental component summary score, the level of the general population was not entirely reached (49.3; 95% CI 49.0–49.6) (32).

Compared to other studies on the cost-effectiveness of psychosocial and psychiatric interventions the gain in quality of life of 0.0568 QALYs in the present study can be classified into the upper range. Out of eleven international publications on psychosocial interventions, only two have reported an increase of more than 0.06 quality-adjusted life-years (33–43). Regarding the incremental expenditure per QALY, additional costs [ranging from 386 € (33) to 53,717 (42)] incurred in each of these studies, which would accordingly apply to an upper right hand quadrant in the cost-effectiveness plane (higher quality of life and higher costs; cost-effectiveness calculations required). Due to the low additional costs (100€ per consultation) and even a decrease of costs in general, our study is the only one in this context qualifying for the dominant quadrant and thereby suggesting acceptability of the intervention as cost-effective.

The integrated care approach of the outreach clinic for clients with mental and psychosocial problems reinforced cross-sectoral cooperation of services underlying different Social Security Codes. This model with its low-threshold counseling and coordinating activities for the above mentioned client group was cost-effective with regard to the relation between healthcare and complementary expenditures and the clients’ quality of life.

## Data Availability Statement

The datasets generated for this study will not be made publicly available. Participants did not give informed consent to open data policy in this study. In addition, anonymization of data in this study sample does not prevent individual participants from being identified on the basis of a specific combination of health and social care utilization.

## Ethics Statement

The studies involving human participants were reviewed and approved by Ethikkommission Schleswig-Holstein [(AZ 131/14 (I)]. The patients/participants provided their written informed consent to participate in this study.

## Author Contributions

The study was designed by AB, TB, TH, DE, and BS. The data were collected by TH, FR, and MA. The analyses were performed by AB and TR. The manuscript was written by SH, AB, TR, and BS.

## Funding

This multi-part project was funded by the Ministry of Social Affairs, Health, Youth, Family and Senior Citizens of the federal state Schleswig-Holstein (Az. 234 - 455.5.004-005/02).

## Conflict of Interest

The authors declare that the research was conducted in the absence of any commercial or financial relationships that could be construed as a potential conflict of interest

The reviewer JS declared a past co-authorship with several of the authors AB and BS to the handling editor.
